# Physician-led prehospital management is associated with reduced mortality in severe blunt trauma patients: a retrospective analysis of the Japanese nationwide trauma registry

**DOI:** 10.1186/s13049-020-00828-4

**Published:** 2021-01-06

**Authors:** Akira Endo, Mitsuaki Kojima, Saya Uchiyama, Atsushi Shiraishi, Yasuhiro Otomo

**Affiliations:** 1 Trauma and Acute Critical Care Center, Tokyo Medical and Dental University Hospital of Medicine, 1-5-45 Yushima, Bunkyo-ku, Tokyo, Japan; 2 Emergency and Critical Care Medicine, Tokyo Women’s Medical University Medical Center East, 2-1-10 Nishiogu, Arakawa-ku, Tokyo, Japan; 3 Department of Professional Development, Tokyo Medical and Dental University Hospital of Medicine, 1-5-45 Yushima, Bunkyo-ku, Tokyo, Japan; 4 Emergency and Trauma Center, Kameda Medical Center, 929 Higashicho, Kamogawa, Chiba, Japan

**Keywords:** Wounds and injuries, Emergency medical service, Prehospital care, Prehospital time, Helicopter emergency medical service, Clinical assessment

## Abstract

**Background:**

Although the results of previous studies suggested the effectiveness of physician-led prehospital trauma management, it has been uncertain because of the limited number of high-quality studies. Furthermore, the advantage of physician-led prehospital management might have been overestimated due to the shortened prehospital time by helicopter transportation in some studies. The present study aimed to evaluate the effect of physician-led prehospital management independent of prehospital time. Also, subgroup analysis was performed to explore the subpopulation that especially benefit from physician-led prehospital management.

**Methods:**

This retrospective cohort study analyzed the data of Japan’s nationwide trauma registry. Severe blunt trauma patients, defined by Injury Severity Score (ISS) ≥16, who were transported directly to a hospital between April 2009 and March 2019 were evaluated. In-hospital mortality was compared between groups dichotomized by the occupation of primary prehospital healthcare provider (i.e., physician or paramedic), using 1:4 propensity score-matched analysis. The propensity score was calculated using potential confounders including patient demographics, mechanism of injury, vital signs at the scene of injury, ISS, and total time from injury to hospital arrival. Subpopulations that especially benefit from physician-led prehospital management were explored by assessing interaction effects between physician-led prehospital management and patient characteristics.

**Results:**

A total of 30,551 patients (physician-led: 2976, paramedic-led: 27,575) were eligible for analysis, of whom 2690 propensity score-matched pairs (physician-led: 2690, paramedic-led: 10,760) were generated and compared. Physician-led group showed significantly decreased in-hospital mortality than paramedic-led group (in-hospital mortality: 387 [14.4%] and 1718 [16.0%]; odds ratio [95% confidence interval] = 0.88 [0.78–1.00], *p* = 0.044). Patients with age < 65 years, ISS ≥25, Abbreviated Injury Scale in pelvis and lower extremities ≥3, and total prehospital time < 60 min were likely to benefit from physician-led prehospital management.

**Conclusions:**

Physician-led prehospital trauma management was significantly associated with reduced in-hospital mortality independent of prehospital time. The findings of exploratory subgroup analysis would be useful for the future research to establish efficient dispatch system of physician team.

**Supplementary Information:**

The online version contains supplementary material available at 10.1186/s13049-020-00828-4.

## Background

Physician-led prehospital management is potentially beneficial in severe trauma patients since physicians are generally allowed to provide broad scope of medical interventions than paramedics. Some previous randomized controlled trials [1, 2] and cohort studies [3–5] suggested beneficial effects of physician-led prehospital trauma management. However, a recent systematic review concluded that evidence supporting the effectiveness of physician-led prehospital management was insufficient because of the limited number of studies with high methodological quality [6].

Notably, some studies evaluating this topic included physician-staffed helicopter emergency medical service (HEMS) which had two major potential advantages: prehospital physician-led management itself and the shortened prehospital transport time. In such studies, the effectiveness of physician-led management might have been overestimated by the benefit of shortened prehospital transport time [7]. Actually, another systematic review [8] reported that benefit of physician-led prehospital trauma management disappeared after excluding helicopter transport as a confounder. Therefore, it would be important to evaluate the benefit of physician-led trauma management independent of prehospital transport time. However, to the best of our knowledge, studies evaluating the independent effect of physician-led trauma management have not been conducted.

The aim of the present study was to evaluate the effect of physician-led prehospital trauma management on patient mortality independent of prehospital time. In addition, we explored the characteristics of patients who were likely to benefit from physician-led prehospital management itself, which would help establishment of future efficient dispatch system of physician-team.

## Methods

### Study design and setting

We conducted a nationwide registry-based retrospective cohort study, wherein we analyzed data from the Japan Trauma Data Bank (JTDB) between April 2009 and March 2019. The details of all trauma patients who suffered a severe injury at any region of the body, with an abbreviated injury scale (AIS) score of ≥3, were registered in the JTDB. During the study period, the JTDB received records from 280 secondary or tertiary emergency hospitals in the country. The database includes information on injury mechanisms, prehospital times (including the times of paramedic dispatch, physician contact, and hospital arrival), patient baseline characteristics (including vital signs at the scene of injury and upon arriving at an emergency department [ED]), procedures performed, and survival status at hospital discharge.

In Japan, the operation of prehospital physician teams, such as dispatch criteria and operating time), varies according to the medical control area. The coverage area also varies largely depending on whether it is an urban or rural area. The physicians are delivered in a car or a helicopter according to the system of the medical control area. They are not always trauma surgeons but are those usually working at an ED and trained to provide basic prehospital trauma management such as assessment with sonography, tracheal intubation, chest drainage, intraosseous infusion, and temporal hemostatic maneuver using a tourniquet. Regarding fluid resuscitation, prehospital blood transfusion is not common in Japan, and only the administration of the crystalloid solution is provided in many cases. In contrast, the medical interventions allowed to Japanese paramedics responding to trauma patients without cardiac arrest are limited to performing spinal motion restriction, external fixation of bone fractures, oxygen administration using a mask, and administration of Ringer’s solution (only to patients with shock).

This study complied with the principles of the 1964 Helsinki Declaration and its later amendments. The Ethics Committee of Tokyo Medical and Dental University approved this study (#2192). The requirement for informed consent from each patient was waived because of the study’s retrospective design and the use of anonymized patient data.

### Study population

Patients who met all of the following criteria were included: (1) patients who aged more than 15 years and suffered blunt injuries of Injury Severity Score (ISS) ≥16, (2) patients who were transferred directly from the scene of injury, and (3) patients whose specific information regarding times of injury, physician contact, and hospital arrival were available. We excluded patients from the analysis if they met at least one of the following criteria: (1) cardiac arrest at the scene of injury, (2) unsalvageable injury defined as AIS = 6, (3) missing data required for analyses (i.e., complete case analysis), and (4) unrealistic or outlier values on prehospital time course, such as time from injury to hospital arrival and time from injury to physician contact, in which outlier values were detected statistically using a single-sample robust linear regression analysis with M estimator [9] and then removed.

### Variables

We collected information on the following items from the JTDB: age, sex, mechanism of injury, year of injury, season of injury, time of injury, time of physician contact, time of hospital arrival, vital signs at the scene of injury (systolic blood pressure, heart rate, and respiratory rate), consciousness level at the scene of injury recorded using the Japan Coma Scale [10] (Supplementary Table 1), vital signs upon hospital arrival (systolic blood pressure, heart rate, and respiratory rate), consciousness level upon hospital arrival recorded using the Glasgow coma scale (GCS), the highest score of AIS values for each region of the body, ISS, and patient survival status at hospital discharge.

Eligible patients were divided into the two groups: patients who received physician-led prehospital management (physician-led group) and the patients who received paramedic-led prehospital management (paramedic-led group). Patients who received physician-led prehospital management were identified by comparing time of physician contact (i.e., the time that the physician started the assessment of the patients) and time of hospital arrival. Season of injury was divided into four categories by quarter, beginning in January. Time of injury was divided into four zones every 6 h, beginning at 0:00. The study outcome was defined by in-hospital mortality.

### Statistical analysis

The present study analyzed non-randomized data in which patient characteristics were not equally distributed between the physician-led and the paramedic-led groups. Considering the unbalanced characteristics between the two groups, we used a propensity score matching analysis [11] to compare the outcome. In this analysis, a logistic regression model was applied to estimate the propensity score for each patient, predicting physician-led prehospital management based on age, sex, mechanism of injury, year of injury, systolic blood pressure and respiratory rate at the scene of injury, consciousness level at the scene of injury, and ISS, in addition to prehospital transport time (from injury onset to hospital arrival). Both the time and season categories of injury were also incorporated into the model. Since the availability of emergency physician or trauma surgeon varies depends on working hours, and the prehospital transport time varies according to weather or road conditions depends on season, these variables could affect the patient outcome in severe trauma. These variables were chosen based on the clinical perspective and subject matter knowledge. The accuracy of a logistic regression model predicting in-hospital mortality with these variables was assessed using C-statistics. Propensity score matching extracted 1:4 matched pairs from the physician-led and paramedic-led groups; this ratio was determined based on the feasibility of match balance and maximum use of patient data. Match balance between the groups was assessed by the absolute standardized mean difference (ASMD); values < 0.1 were considered acceptable [12]. The caliper width was set as the standard deviation of the logit-transformed propensity score multiplied by 0.1 to achieve well-matched balance between the two groups. The chi-square test was used for intergroup comparison in the propensity score-matched cohort. As a sensitivity analysis, we also evaluated the effectiveness of physician-led prehospital management using a multivariate logistic regression model in an overall study cohort (i.e., not the propensity score-matched cohort). In this model, the aforementioned variables used in the propensity score calculation were used as the covariates. Multicollinearity was assessed by the variance inflation factor, with the tolerance value set at < 2.

Subgroup analysis was performed in the propensity score–matched cohort to explore potential patients who were likely to benefit from physician-led prehospital management. We evaluated the *p* values for the interaction between physician-led prehospital trauma management and the following dichotomized categories for in-hospital mortality: age (< 65 vs. ≥65), sex (male vs. female), blood pressure at the scene of injury (< 90 mmHg vs. ≥90 mmHg), shock index defined by the heart rate/systolic blood pressure ratio (< 1 vs. ≥1), presence or absence of coma (defined by Japan Coma Scale > 30 at the scene of injury), ISS (< 25 vs. ≥25), the highest AIS scores on the head, chest, abdomen, and pelvis and lower extremities (< 3 vs. ≥3), and the time lapse between the time of injury and the time of hospital arrival (< 60 min vs. ≥60 min).

Descriptive statistics were reported as counts and percentages for categorical variables and medians and the 25th–75th percentiles for numeric or ordered variables. Predictive statistics were reported as odds ratios (ORs) and 95% confidence intervals (CIs). The level of significance was defined as two-sided *p* < 0.05 for all statistical analyses. All analyses were performed using R 3.5.3 (R Foundation for Statistical Computing, Vienna, Austria) with add-on packages of “Matching [13]” for propensity score matching and “robustbase [14]” for robust regression analysis.

## Results

**Fig. 1:**
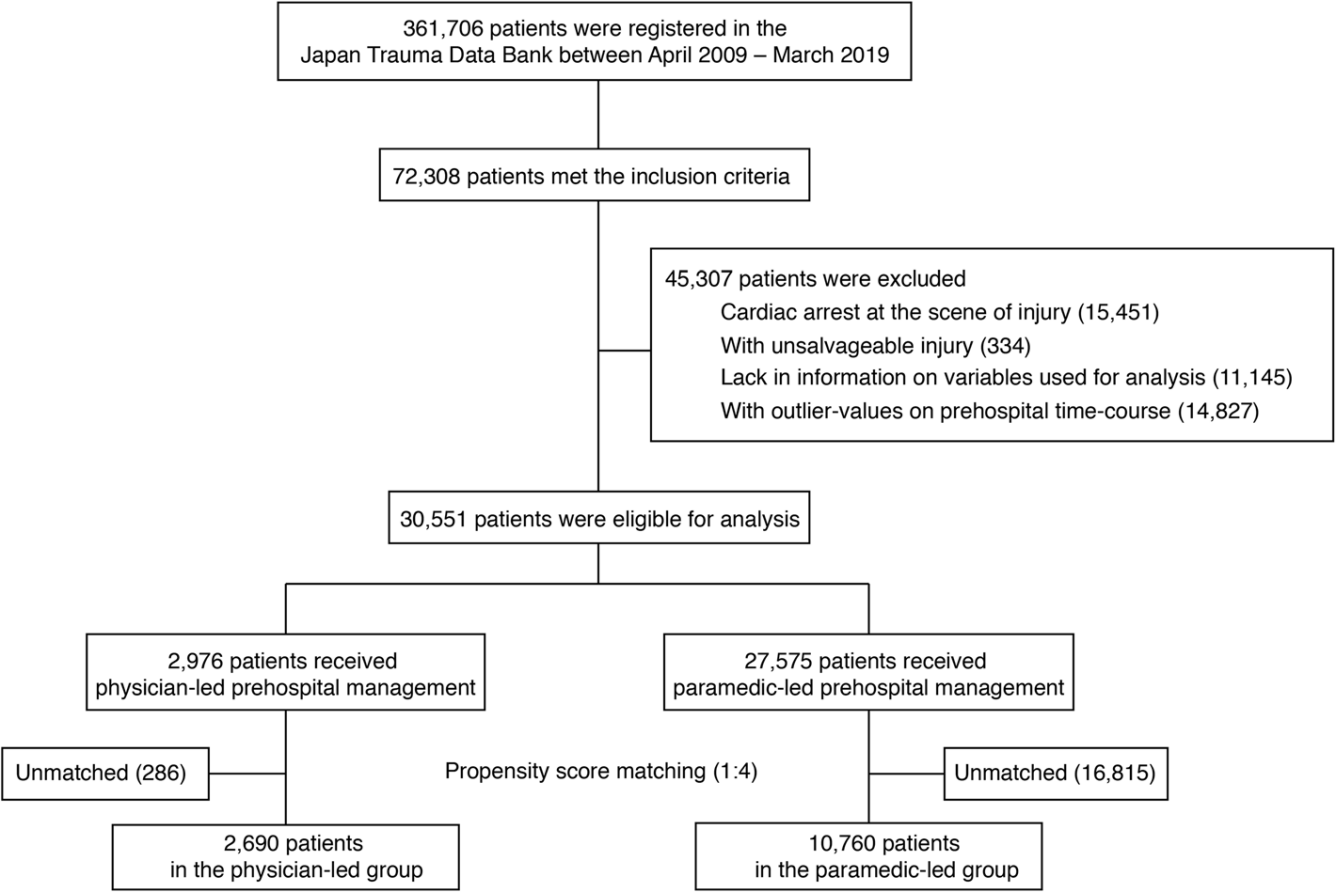
Flow diagram of the process of patient selection

A flow diagram of the patient selection process is presented in Fig. 1. A total of 30,551 patients were eligible for analysis based on the inclusion and exclusion criteria. Of them, 2976 patients (9.7%) received physician-led prehospital management. The major baseline characteristics of the overall study cohort are summarized on the left part of the Table 1. All the variables of patient characteristics are summarized on the left part of the Supplementary Table 2. Physician-led prehospital management was more likely to be provided during daytime (between 6:00 and 17:59) than during nighttime. The difference in the values of ISS between two groups suggested that patients in the physician-led group suffered more severe injuries than those in the paramedic-led group. In-hospital mortality was observed in 453 (15.2%) patients in the physician-led group and in 3385 (12.3%) patients in the paramedic-led group.

**Table 1:** Characteristics of the patients before and after propensity score matching (All variables)

Variables	Overall study cohort			Propensity score–matched cohort		
	Physician-led group (*n* = 2976)	Paramedic-led group (*n* = 27,575)	ASMD	Physician-led group (*n* = 2690)	Paramedic-led group (*n* = 10,760)	ASMD
Age, years, median (IQR)	62 (42, 74)	63 (43, 75)	0.011	62 (41, 74)	62 (43, 75)	0.024
Females, n (%)	855 (28.7)	8091 (29.3)	0.013	760 (28.3)	3167 (29.4)	0.026
Year of injury, n (%)			0.136			0.087
2009–2013	1089 (36.6)	10.768 (39.0)		1086 (40.4)	4081 (37.9)	
2014–2019	1887 (63.4)	16,807 (61.0)		1604 (59.6)	6679 (62.1)	
Time of injury, n (%)			0.332			0.008
Daytime	2285 (76.8)	17,019 (61.7)		2017 (75.0)	8074 (75.0)	
Nighttime	691 (23.2)	10,556 (38.3)		673 (25.0)	2686 (25.0)	
Mechanism of injury, n (%)			0.357			0.041
Traffic accident	1662 (55.8)	14,285 (51.8)		1482 (55.1)	5948 (55.3)	
Fall from height	522 (17.5)	3475 (12.6)		455 (16.9)	1852 (17.2)	
Fall (others)	552 (18.5)	8344 (30.3)		541 (20.1)	2180 (20.3)	
Others	240 (8.1)	1471 (5.3)		212 (7.9)	780 (7.2)	
Vital signs at the scene of injury, median (IQR)						
Systolic blood pressure, mmHg	132 (110, 159)	135 (112, 160)	0.056	134 (113, 156)	134 (112, 158)	0.032
Heart rate^a^, beats/min	84 (72, 100)	84 (72, 98)	0.071	84 (70, 100)	84 (72, 100)	0.012
Respiratory rate, breaths/min	24 (19, 28)	20 (18, 24)	0.167	21 (18, 26)	21 (18, 26)	< 0.001
Japan Coma Scale at the scene of injury, n (%)			0.226			0.020
0 (alert)	774 (26.0)	7855 (28.5)		721 (26.8)	2898 (26.9)	
300 (deep coma)	374 (12.6)	2484 (9.0)		303 (11.3)	1263 (1.7)	
The highest score of AIS, median (IQR)						
Head^a^	3 (0, 4)	3 (0, 4)	0.007	3 (0, 4)	3 (0, 4)	0.017
Face^a^	0 (0, 0)	0 (0, 0)	0.044	0 (0, 0)	0 (0, 0)	0.026
Neck^a^	0 (0, 0)	0 (0, 0)	0.014	0 (0, 0)	0 (0, 0)	0.036
Chest^a^	3 (0, 4)	0 (0, 3)	0.251	3 (0, 4)	2 (0, 4)	0.048
Abdomen^a^	0 (0, 0)	0 (0, 0)	0.12	0 (0, 0)	0 (0, 0)	0.017
Spine^a^	0 (0, 2)	0 (0, 2)	0.027	0 (0, 2)	0 (0, 2)	0.005
Upper extremities^a^	0 (0, 2)	0 (0, 1)	0.124	0 (0, 2)	0 (0, 2)	0.036
Pelvis and lower extremities^a^	0 (0, 2)	0 (0, 2)	0.166	0 (0, 2)	0 (0, 2)	0.044
Surface^a^	0 (0, 0)	0 (0, 0)	0.02	0 (0, 0)	0 (0, 0)	< 0.001
ISS, median (IQR)	25 (18, 33)	21 (17, 27)	0.279	24 (17, 30)	24 (17, 29)	0.007
Prehospital time-course, min, median (IQR)						
Injury to physician contact^a^	40 (30, 55)	46 (35, 61)	0.331	39 (29, 53)	53 (39, 72)	0.719
Injury to ED arrival	56 (42, 73)	44 (34, 58)	0.477	54 (41, 68)	50 (38, 70)	0.006
Transporter, n (%)			1.741			1.478
Air ambulance^a^	971 (32.6)	1430 (5.2)		829 (30.8)	1078 (10.0)	
Ground ambulance^a^	2005 (67.4)	26,113 (94.7)		1861 (69.2)	9667 (89.8)	
Others^a^	0 (0)	32 (0.1)		0 (0)	15 (0.1)	
Intubation in the prehospital settings^a^, n (%)	248 (8.4)	250 (0.9)	0.361	199 (7.5)	192 (1.8)	0.271

*Abbreviations*: *ASMD* Absolute standardized mean difference, *IQR* Interquartile range, *AIS* Abbreviated injury scale, *ISS* Injury severity score, *ED* Emergency department

^a^These variables were not included in the model for propensity score estimation

The variables used for propensity score estimation had high accuracy for predicting in-hospital mortality with C-statistics of 0.87. Via the matching process, a total of 2690 propensity score-matched pairs (2690 and 10,760 patients per physician-led group and paramedic-led group, respectively) were generated. All the ASMD values of the adjusted variables for the severity adjustment were < 0.1, indicating a well-matched balance (the right part of the Table 1 and Supplementary Table 2). Time lapse from the time of injury to physician contact was shorter in the physician-led group than in the paramedic-led group. In-hospital mortality was observed in 387 (14.4%) patients in the physician-led group and in 1718 (16.0%) patients in the paramedic-led group. A significantly reduced in-hospital mortality rate was observed in the physician-led group in the propensity score-matched population (OR = 0.88, 95% CI, 0.78–1.00; *p* = 0.044). In the sensitivity analysis conducted with the overall study population using logistic regression analysis, the variance inflation factors for all the variables were less than 2, which eliminated the issue of multicollinearity in the model. The result also showed the significant association between physician-led prehospital treatment and reduced in-hospital mortality (adjusted OR =0.85, 95% CI,0.74–0.97; *p* = 0.015).

**Fig. 2:**
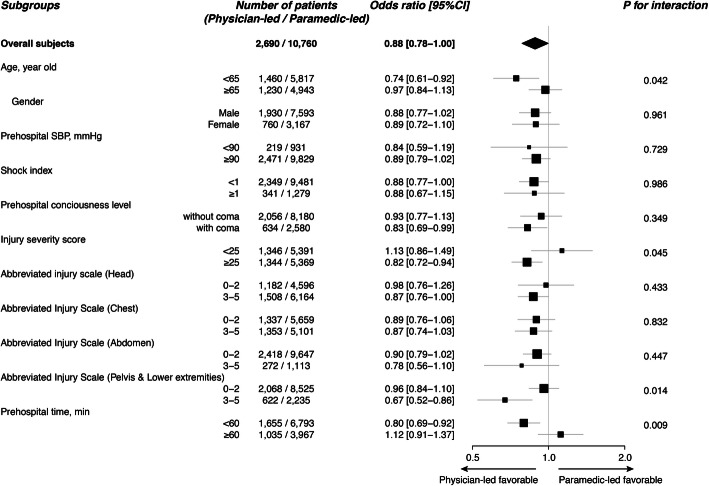
Subgroup analysis for the effect of physician-led prehospital trauma management on in-hospital mortality

The results of subgroup analysis are summarized in Fig. 2. The *p* values for interaction between physician-led prehospital management and the following dichotomized variables were statistically significant: age (< 65 vs. ≥65), ISS (< 25 vs. ≥25), AIS scores on pelvis and lower extremities (< 3 vs. ≥3), and the total prehospital time (< 60 min vs. ≥60 min).

## Discussion

The present study demonstrated that physician-led prehospital trauma care, compared to paramedic-led trauma care, was significantly associated with reduction in mortality independent of prehospital transport time. Some previous studies showing the superiority of physician-led prehospital trauma management might have been largely benefitted by the shortened prehospital transport time of HEMS [15, 16], in addition to the independent effect of physician-led prehospital care. To the best of our knowledge, this is the first large-scale study to show the effectiveness of physician-led prehospital trauma care itself. Furthermore, exploratory subgroup analysis revealed the specific subpopulations that might be likely to benefit from physician-led prehospital management, which would be potentially useful for the establishment of the dispatch criteria in future.

Since the present study was a retrospective study analyzing the existing trauma registry, patient characteristics were not equally distributed between the physician-led and the paramedic-led groups. To control the unbalanced characteristics between two groups, we used a propensity score matching analysis [11] in which patients with similar likelihood for the intervention (i.e., physician-led prehospital management) could be compared considering potential confounders available in the JTDB. As comparing patients with extremely low or high probability for the intervention was not reasonable, inverse probability of treatment weighting method was not used in the primary analysis. The result of sensitivity analysis using logistic regression analysis suggested the robustness of the result of propensity score matching analysis. However, because the JTDB was not a registry specialized for prehospital care, several important information on this topic, such as medical control area, physical assessment results, and delivered treatments, were not available, which could have led to the issue of residual confounding. Effectiveness of physician-led prehospital trauma care would vary according to the location (urban or rural), and the results of the physical assessment by paramedic would be necessary to establish optimal dispatch criteria of physician-team. Further studies taking these variables into account would be required to confirm our result.

Several theoretical advantages of physician-led over paramedic-led prehospital management of severe trauma cases, in addition to the broader scope of medical interventions, should be considered. Physicians’ interventions were reported to have a higher success rate than those performed by paramedics; for example, a previous study showed a correspondingly higher rate of achieving successful advanced airway management [17–19], which prevents secondary brain injury [20]. Moreover, physicians can make precise and flexible clinical decisions following the latest trauma management strategy rather than uniform simplified management, such as introduction of restrictive fluid management based on strategic permissive hypotension [21]. Regrettably, the JTDB lacks detailed information on the treatments provided by physicians in prehospital settings, which prevented us from specifying the interventions or decision-making processes that contributed to the decreased mortality in the present study.

Meanwhile, the results of the subgroup analysis in the present study suggested the potential subpopulations who were more likely to benefit from physician-led prehospital management: patient who had age < 65 years, severe injuries with ISS ≥25, injury with AIS ≥3 in the pelvis or lower extremities, and total transportation time < 60 min. A previous study assessing the characteristics of geriatric trauma patients reported a positive linear relationship between age and mortality risk [22], suggesting that the effects of any treatment provided by a physician in prehospital settings might be smaller in older patients. Regarding the ISS, our result was consistent with previous studies showing the effectiveness of physician-led prehospital management especially in severe trauma patients [23, 24]. Notably, patients who suffered severe injury in the pelvis or lower extremities were more likely to benefit from physician-led prehospital management. This could be partially explained by the nature of the procedures performed in the prehospital settings. Interventions that can be provided in the prehospital setting are generally limited to simple procedures, including the use of a tourniquet or resuscitative endovascular balloon occlusion of the aorta, while multiple and complicated intra thoracic or abdominal organ injuries cannot be repaired anatomically. Thus, treatments in prehospital settings might have been provided as bridging therapies until definitive care that can be provided after hospital arrival. This hypothesis could also explain the result that total prehospital time was significantly associated with the effectiveness of physician-led prehospital management. Prolonged prehospital transport time might have reduced the effect of physician-led prehospital bridging treatments. Since the matching of patient backgrounds was insufficient in the subgroup analysis, the results should be interpreted as exploratory. However, the findings can serve as a basis for a future study establishing optimal indication for dispatching prehospital physician-teams.

The strength of the present study was that we analyzed a large-scale nationwide trauma registry. Although a previous study [4] showed significant association between physician-led prehospital management and reduction in mortality, the association did not reach significant level in the model that considered prehospital time possibly due to smaller sample size than ours. Clinically relevant potential confounders were taken into account as far as possible. However, there were several limitations to this study that should be acknowledged. The issue of residual confounding was unavoidable due to the retrospective nature of this study. Detailed information on the prehospital settings was not available in the JTDB. These limitations have already been discussed. Physician dispatch criteria did not follow standardized protocols. The consciousness level of a patient at the scene of the injury was recorded using the JCS, not the GCS, which was not in global use. The evaluated population would be older than those in other countries because Japan is the most aged country. Furthermore, medical interventions that paramedics can provide vary across countries. The regional difference in the demographic and medical system limited the generalizability of the results, and the conclusion would not always be applicable in different countries. Despite these limitations, to the best of our knowledge, this was the first largescale retrospective cohort study that showed the independent survival benefit of physician-led prehospital trauma management. Future studies considering detailed information on prehospital settings, such as mechanisms of injury, location, results of physical assessment by paramedics, and delivered medical interventions, would be needed for establishing optimal dispatch criteria for a prehospital physician-team.

## Conclusions

This large-scale retrospective cohort study showed a significant association between patient survival and physician-led prehospital trauma management independent of prehospital transport time. Patient who had age < 65 years, severe injuries with ISS ≥25, injury with AIS ≥3 in the pelvis or lower extremities, and total transportation time < 60 min might benefit from physician-led prehospital management.

## Supplementary Information

**Additional file 1: Table S1.** Comparison between Japan Coma Scale and Glasgow Coma Scale.

**Additional file 2: Table S2.** Characteristics of the patients before and after propensity score matching (All variables).

## Data Availability

An overview of the Japan Trauma Data Bank (JTDB) is available at http://www.jtcr-jatec.org/traumabank/index.htm. The detailed data in the JTDB that support the findings of this study are available from Japan Trauma Care and Research but restrictions apply to the availability of these data, which were used under license for the current study, and hence are not publicly available.

## Abbreviations

AIS: Abbreviated injury scale
ASMD: Absolute standardized mean difference
CI: Confidence interval
ED: Emergency department
GCS: Glasgow Coma Scale
HEMS: Helicopter emergency medical service
ISS: Injury severity score
JTDB: Japan Trauma Data Bank
OR: Odds ratio
