# Supplementary material for: Physician-led prehospital management is associated with reduced mortality in severe blunt trauma patients: a retrospective analysis of the Japanese nationwide trauma registry
Source: Scand J Trauma Resusc Emerg Med. 2021 Jan 6;29:9. doi: 10.1186/s13049-020-00828-4 (PMC7789566; doi:10.1186/s13049-020-00828-4)

| **Supplementary table 1. Comparison between Japan Coma Scale and Glasgow Coma Scale** | | | |
| --- | --- | --- | --- |
| Japan Coma Scale | Glasgow Coma Scale | | |
|  | Eye opening | Verbal | Motor |
| 0: Alert | 4 | 5 | 6 |
| 1: Open eye spontaneously and almost alert | 4 | 5 | 6 |
| 2: Open eye spontaneously but disoriented | 4 | 4 | 6 |
| 3: Open eye spontaneously but unable to recall name and date of birth | 4 | Unspecified | Unspecified |
| 10: Open eye to speech | 3 | Unspecified | Unspecified |
| 20: Open eye to stimuli | 3 | Unspecified | Unspecified |
| 30: Open eye to repeated painful stimuli | 2 | Unspecified | Unspecified |
| 100: Cannot open eye but moves to localized pain | 1 | Unspecified | 5 |
| 200: Cannot open eye but responds to painful stimuli with slight movement | 1 | Unspecified | 4, 3, or 2 |
| 300: No response | 1 | 1 | 1 |
| Glasgow Coma Scale is defined by the following three-axis scale:  [Eye opening response]  4: Spontaneously, 3: To speech, 2: To pain, 1: No response  [Best verbal response]  5: Oriented, 4: confused, 3: Inappropriate words, 2: Incomprehensible sounds, 1: No response  [Best motor response]  6: Obeys commands, 5: Moves to localized pain, 4: Flexion withdrawal from pain, 3: Abnormal flexion (decorticate), 2: Abnormal extension (decerebrate), 1: No response | | | |


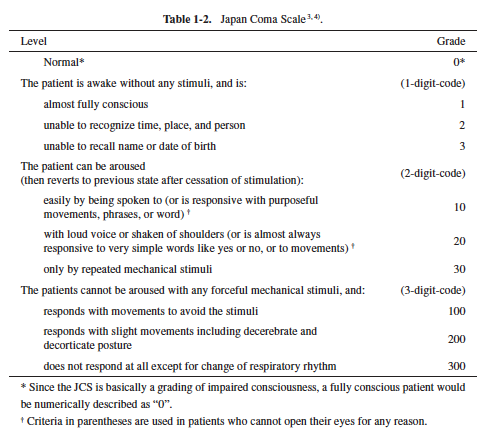

Supplement: Supplementary file 1 — Additional file 1: Table S1. Comparison between Japan Coma Scale and Glasgow Coma Scale. [file 13049_2020_828_MOESM1_ESM.docx]
