# Supplementary material for: Physician-led prehospital management is associated with reduced mortality in severe blunt trauma patients: a retrospective analysis of the Japanese nationwide trauma registry
Source: Scand J Trauma Resusc Emerg Med. 2021 Jan 6;29:9. doi: 10.1186/s13049-020-00828-4 (PMC7789566; doi:10.1186/s13049-020-00828-4)
Supplement: Supplementary file 2 — Additional file 2: Table S2. Characteristics of the patients before and after propensity score matching (All variables). [file 13049_2020_828_MOESM2_ESM.docx]

| **Supplementary table 2. Characteristics of the patients before and after propensity score matching (All variables)** | | | | | | | |
| --- | --- | --- | --- | --- | --- | --- | --- |
| Variables | | Overall study cohort | | | Propensity score–matched cohort | | |
|  |  | Physician-led group  (n = 2,976) | Paramedic-led group  (n = 27,575) | ASMD | Physician-led group  (n = 2,690) | Paramedic-led group  (n = 10,760) | ASMD |
| Age, years, median (IQR) | | 62 (42, 74) | 63 (43, 75) | 0.011 | 62 (41, 74) | 62 (43, 75) | 0.024 |
| Females, n (%) | | 855 (28.7) | 8,091 (29.3) | 0.013 | 760 (28.3) | 3,167 (29.4) | 0.026 |
| Year of injury, n (%) | |  |  | 0.136 |  |  | 0.087 |
|  | 2009 | 120 (4.0) | 1,326 (4.8) |  | 120 (4.5) | 476 (4.4) |  |
|  | 2010 | 159 (5.3) | 1,634 (5.9) |  | 158 (5.9) | 601 (5.6) |  |
|  | 2011 | 194 (6.5) | 2,157 (7.8) |  | 194 (7.2) | 725 (6.7) |  |
|  | 2012 | 300 (10.1) | 2,632 (9.5) |  | 300 (11.2) | 1,154 (10.7) |  |
|  | 2013 | 316 (10.6) | 3,019 (10.9) |  | 314 (11.7) | 1,125 (10.5) |  |
|  | 2014 | 376 (12.6) | 3,176 (11.5) |  | 354 (13.2) | 1,322 (12.3) |  |
|  | 2015 | 390 (13.1) | 3,489 (12.7) |  | 365 (13.6) | 1,391 (12.9) |  |
|  | 2016 | 416 (14.0) | 3,066 (11.1) |  | 328 (12.2) | 1,437 (13.4) |  |
|  | 2017 | 364 (12.2) | 3,196 (11.6) |  | 276 (10.3) | 1,244 (11.6) |  |
|  | 2018 | 310 (10.4) | 3,463 (12.6) |  | 257 (9.6) | 1,177 (10.9) |  |
|  | 2019 | 31 (1.0) | 417 (1.5) |  | 24 (0.9) | 108 (1.0) |  |
| Season of injury, n (%) | |  |  | 0.054 |  |  | 0.010 |
|  | January–March | 641 (21.5) | 6,386 (23.2) |  | 585 (21.7) | 2,310 (21.5) |  |
|  | April–June | 734 (24.7) | 6,478 (23.5) |  | 667 (24.8) | 2,645 (24.6) |  |
|  | July–September | 781 (26.2) | 6,836 (24.8) |  | 699 (26.0) | 2,822 (26.2) |  |
|  | October–December | 820 (27.6) | 7,875 (28.6) |  | 739 (27.5) | 2,983 (27.7) |  |
| Time of injury, n (%) | |  |  | 0.332 |  |  | 0.008 |
|  | 0:00–5:59 | 246 (8.3) | 4,041 (14.7) |  | 240 (8.9) | 963 (8.9) |  |
|  | 6:00–11:59 | 1,139 (38.3) | 8,374 (30.4) |  | 997 (37.1) | 4,028 (37.4) |  |
|  | 12:00–17:59 | 1,146 (38.5) | 8,645 (31.4) |  | 1,020 (37.9) | 4,046 (37.6) |  |
|  | 18:00–23:59 | 445 (15.0) | 6,515 (23.6) |  | 433 (16.1) | 1,723 (16.0) |  |
| Mechanism of injury, n (%) | |  |  | 0.357 |  |  | 0.041 |
|  | Traffic accident (Car, driver) | 389 (13.1) | 2,576 (9.3) |  | 324 (12.0) | 1,309 (12.2) |  |
|  | Traffic accident (Car, Navigator) | 72 (2.4) | 528 (1.9) |  | 65 (2.4) | 259 (2.4) |  |
|  | Traffic accident (Car, rear seat) | 62 (2.1) | 399 (1.4) |  | 49 (1.8) | 212 (2.0) |  |
|  | Traffic accident (Motorcycle, driver) | 418 (14.0) | 4,052 (14.7) |  | 393 (14.6) | 1547 (14.4) |  |
|  | Traffic accident (Motorcycle, rear seat) | 17 (0.6) | 123 (0.4) |  | 15 (0.6) | 52 (0.5) |  |
|  | Traffic accident (Bicycle) | 279 (9.4) | 3,038 (11.0) |  | 264 (9.8) | 1,023 (9.5) |  |
|  | Traffic accident (Pedestrian) | 410 (13.8) | 3,468 (12.6) |  | 358 (13.3) | 1,496 (13.9) |  |
|  | Traffic accident (Others) | 15 (0.5) | 101 (0.4) |  | 14 (0.5) | 50 (0.5) |  |
|  | Train-related injury | 25 (0.8) | 141 (0.5) |  | 22 (0.8) | 91 (0.8) |  |
|  | Fall from height | 522 (17.5) | 3,475 (12.6) |  | 455 (16.9) | 1,852 (17.2) |  |
|  | Fall from stairs | 364 (12.2) | 4,278 (15.5) |  | 353 (13.1) | 1,382 (12.8) |  |
|  | Ground-level fall | 188 (6.3) | 4,066 (14.7) |  | 188 (7.0) | 798 (7.4) |  |
|  | Others | 215 (7.2) | 1,330 (4.8) |  | 190 (7.1) | 689 (6.4) |  |
| Vital signs at the scene of injury, median (IQR) | |  |  |  |  |  |  |
|  | Systolic blood pressure, mmHg | 132 (110, 159) | 135 (112, 160) | 0.056 | 134 (113, 156) | 134 (112, 158) | 0.032 |
|  | Heart rate*, beats/min | 84 (72, 100) | 84 (72, 98) | 0.071 | 84 (70, 100) | 84 (72, 100) | 0.012 |
|  | Respiratory rate, breaths/min | 24 (19, 28) | 20 (18, 24) | 0.167 | 21 (18, 26) | 21 (18, 26) | <0.001 |
| Japan Coma Scale at the scene of injury, n (%) | |  |  | 0.226 |  |  | 0.020 |
|  | 0 (alert) | 774 (26.0) | 7,855 (28.5) |  | 721 (26.8) | 2,898 (26.9) |  |
|  | 1 | 526 (17.7) | 5,395 (19.6) |  | 489 (18.2) | 1,944 (18.1) |  |
|  | 2 | 249 (8.4) | 3,478 (12.6) |  | 242 (9.0) | 952 (8.8) |  |
|  | 3 | 221 (7.4) | 2,275 (8.3) |  | 209 (7.8) | 824 (7.7) |  |
|  | 10 | 299 (10.0) | 1,987 (7.2) |  | 256 (9.5) | 1,018 (9.5) |  |
|  | 20 | 61 (2.0) | 585 (2.1) |  | 57 (2.1) | 226 (2.1) |  |
|  | 30 | 98 (3.3) | 678 (2.5) |  | 82 (3.0) | 318 (3.0) |  |
|  | 100 | 179 (6.0) | 1,372 (5.0) |  | 157 (5.8) | 643 (6.0) |  |
|  | 200 | 195 (6.6) | 1,466 (5.3) |  | 174 (6.5) | 674 (6.3) |  |
|  | 300 (deep coma) | 374 (12.6) | 2,484 (9.0) |  | 303 (11.3) | 1,263 (1.7) |  |
| The highest score of AIS, median (IQR) | |  |  |  |  |  |  |
|  | Head* | 3 (0, 4) | 3 (0, 4) | 0.007 | 3 (0, 4) | 3 (0, 4) | 0.017 |
|  | Face* | 0 (0, 0) | 0 (0, 0) | 0.044 | 0 (0, 0) | 0 (0, 0) | 0.026 |
|  | Neck* | 0 (0, 0) | 0 (0, 0) | 0.014 | 0 (0, 0) | 0 (0, 0) | 0.036 |
|  | Chest* | 3 (0, 4) | 0 (0, 3) | 0.251 | 3 (0, 4) | 2 (0, 4) | 0.048 |
|  | Abdomen* | 0 (0, 0) | 0 (0, 0) | 0.12 | 0 (0, 0) | 0 (0, 0) | 0.017 |
|  | Spine* | 0 (0, 2) | 0 (0, 2) | 0.027 | 0 (0, 2) | 0 (0, 2) | 0.005 |
|  | Upper extremities* | 0 (0, 2) | 0 (0, 1) | 0.124 | 0 (0, 2) | 0 (0, 2) | 0.036 |
|  | Pelvis and lower extremities* | 0 (0, 2) | 0 (0, 2) | 0.166 | 0 (0, 2) | 0 (0, 2) | 0.044 |
|  | Surface* | 0 (0, 0) | 0 (0, 0) | 0.02 | 0 (0, 0) | 0 (0, 0) | <0.001 |
| ISS, median (IQR) | | 25 (18, 33) | 21 (17, 27) | 0.279 | 24 (17, 30) | 24 (17, 29) | 0.007 |
| Prehospital time-course, min, median (IQR) | |  |  |  |  |  |  |
|  | Injury to paramedic contact* | 8 (6, 11) | 7 (5, 9] | 0.099 | 8 (6, 11) | 7 (5, 10) | 0.035 |
|  | Paramedic contact to ED arrival* | 46 (34, 62) | 36 (27, 49) | 0.323 | 45 (33, 58) | 42 (30, 59) | 0.010 |
|  | Injury to ED arrival | 56 (42, 73) | 44 (34, 58) | 0.477 | 54 (41, 68) | 50 (38, 70) | 0.006 |
|  | Injury to physician contact* | 40 (30, 55) | 46 (35, 61) | 0.331 | 39 (29, 53) | 53 (39, 72) | 0.719 |
| Transporter, n (%) | |  |  | 1.741 |  |  | 1.478 |
|  | Air ambulance* | 971 (32.6) | 1,430 (5.2) |  | 829 (30.8) | 1,078 (10.0) |  |
|  | Ground ambulance* | 2,005 (67.4) | 26,113 (94.7) |  | 1,861 (69.2) | 9,667 (89.8) |  |
|  | Others* | 0 (0) | 32 (0.1) |  | 0 (0) | 15 (0.1) |  |
| Vital signs upon ED arrival, median (IQR) | |  |  |  |  |  |  |
| Systolic blood pressure*, mmHg | | 132 [112, 155] | 136 [114, 160] | 0.12 | 134 [113, 156] | 134 [112, 158] | 0.032 |
| Heart rate*, beats/min | | 84 [70, 100] | 83 [71, 98] | 0.07 | 84 [70, 100] | 84 [72, 100] | 0.012 |
| Respiratory rate*, breaths/min | | 21 [18, 26.] | 20 [18, 25] | 0.055 | 21 [18, 26] | 21 [18, 26] | <0.001 |
| GCS upon ED arrival*, median (IQR) | | 14 [9, 15] | 14 [11, 15] | 0.191 | 14 [9, 15] | 14 [10. 15] | 0.035 |
| Intubation in the prehospital settings*, n (%) | | 248 (8.4) | 250 (0.9) | 0.361 | 199 (7.5) | 192 (1.8) | 0.271 |
| *These variables were not included in the model for propensity score estimation.  Abbreviations: ASMD, absolute standardized mean difference; IQR, interquartile range; AIS, abbreviated injury scale; ISS, injury severity score; ED, emergency department; GCS, Glasgow Coma Scale. | | | | | | | |
